# Letting the patients speak: an in-depth, qualitative research-based investigation of factors relevant to health-related quality of life in real-world patients with hereditary angioedema using subcutaneous C1 inhibitor replacement therapy

**DOI:** 10.1186/s13223-021-00550-5

**Published:** 2021-06-27

**Authors:** John Anderson, Donald S. Levy, William Lumry, Patricia Koochaki, Sally Lanar, H. Henry Li

**Affiliations:** 1 Clinical Research Center of Alabama, Birmingham, AL USA; 2 UC Irvine, Orange, CA USA; 3 AARA Research Center, Dallas, TX USA; 4 ICON, Cincinnati, OH USA; 5 ICON, Lyon, France; 6 Institute for Asthma and Allergy, Chevy Chase, MD USA

**Keywords:** Subcutaneous C1-inhibitor, Hereditary angioedema, Health-related quality of life, Productivity, HAEGARDA, Qualitative research

## Abstract

**Background:**

While many studies of effective hereditary angioedema (HAE) therapy have demonstrated improved health-related quality of life (HRQoL) using validated instruments, specific reasons behind the improved scores have never been investigated using qualitative methods. A non-interventional, qualitative research study was designed to investigate the reasons for improvements in HRQoL while using effective prophylaxis, in this case subcutaneous C1INH (C1INH[SC]) replacement therapy.

**Methods:**

Adult patients with HAE-C1INH type 1 or 2 who had been using C1INH(SC) for ≥ 3 consecutive months were recruited through four HAE specialty practices in the US to participate in a 60-min phone interview performed by a trained qualitative research specialist (ICON plc) using a semi-structured interview guide with open-ended questions developed with the Angioedema Quality of Life (AE-QoL) items in mind. Interview transcripts were analyzed using thematic analysis methods to identify concepts (specific symptoms/impacts) and themes (higher-level categories grouping related concepts). A cross-mapping exercise was performed between interview-identified concepts and items included in the AE-QoL.

**Results:**

Fourteen patients were interviewed and included in the analysis (age range, 28–82 years [mean 47.5 years]; 64% female; 93% white). In 10 interviews, patients mentioned having no or nearly no HAE attacks, no longer feeling limited by HAE, less HAE-related anxiety/worry and depression, an improved ability to travel, fewer emergency room/hospital visits, and ease of administration of C1INH(SC), including not requiring assistance from others. Other commonly expressed concepts included: increased feelings of confidence, independence, optimism, and normalcy; less absence from work/school; better productivity; improved sleep and energy; healthier family relationships; and improved cognition. While all AE-QoL items emerged from patient interviews, a number of identified concepts were not addressed by the AE-QoL, including sensitivity to various potential attack-triggers (e.g., stress/anxiety, sports), attack frequency, not having to cancel social plans, improvements in ability to perform day-to-day tasks, and a lower burden from medical visits.

**Conclusions:**

From these interviews, a large number of common themes and concepts emerged: a greater sense of freedom and normalcy, increased productivity, and improved interpersonal relationships while using convenient and effective prophylaxis. These findings provide insights into real-world experiences and the many facets of HRQoL that are important to patients with HAE-C1INH.

**Supplementary Information:**

The online version contains supplementary material available at 10.1186/s13223-021-00550-5.

## Background

Hereditary angioedema (HAE) with C1-inhibitor deficiency (HAE-C1INH) is a rare genetic disorder in which there exists either a deficiency of C1INH protein (HAE-C1INH type 1) or production of C1INH that is dysfunctional (HAE-C1INH type 2). The clinical manifestations of these pathologies are the same and include ongoing, generally unpredictable edema attacks that typically affect the face, extremities, abdomen, and the upper airways. Multiple factors contribute to profound health-related quality of life (HRQoL) burdens in patients with HAE-C1INH, including the chronic, lifelong nature of the disease, the pain and disfigurement that accompanies attacks, anxiety over potentially fatal laryngeal attacks, and disruptions in productivity and social interactions.

The treatment landscape for HAE-C1INH has undergone tremendous expansion over recent years with numerous regulatory approvals in the US and many international countries for drugs specifically designed to treat or prevent attacks. Medications for treating attacks (on-demand treatment) include intravenously (IV) administered human plasma-derived C1INH (Berinert^®^/CSL Behring) and recombinant C1INH (Ruconest^®^; Pharming Healthcare) and two subcutaneously (SC) administered products: ecallantide (Kalbitor^®^; Shire), a plasma kallikrein inhibitor; and icatibant (Firazyr^®^; Shire), a bradykinin B-2 receptor antagonist. Therapies for preventing attacks (prophylaxis) include IV plasma-derived C1INH (Cinryze^®^, Shire), SC plasma-derived C1INH (HAEGARDA^®^, CSL Behring), and the SC monoclonal antibody lanadelumab (TAKHZYRO™/Takeda [formerly Shire]).

With the advent of highly effective therapies for the management of HAE-C1INH, treatment guidelines have increasingly emphasized improved HRQoL as an important aspect of disease management [1–3]. Accordingly, measurement of HRQoL has become a common and important element of HAE-C1INH clinical trials. As further evidence of the increasing importance of HRQoL in the field of HAE-C1INH, disease-specific HRQoL tools continue to be developed, including the Angioedema Quality of Life Questionnaire (AE-QoL) [4] and the Hereditary Angioedema Quality of Life Questionnaire (HAE-QoL) [5, 6]. The AE-QoL was developed with input from patients with various types of angioedema including both bradykinin-mediated (e.g., HAE-C1INH) and histamine-mediated angioedema [4]; thus, it is more disease-specific than generic HRQoL instruments but may not address all aspects of HAE-related HRQoL. The HAE-QoL is a targeted HAE-specific instrument, based on interviews from adult patients with HAE and initially validated in Europe [5]. HRQoL data obtained from studies using any validated questionnaire, both generic and disease-specific, can provide important information on the effectiveness of treatments and allow comparisons of treatment effectiveness on HRQoL over time. In addition, as additional information about patients’ experiences and HRQoL becomes available, additional concepts important to patients can sometimes be elicited and lead to the development of new questionnaires or adaptation of existing questionnaires to include the new concepts.

Qualitative research is a field of study that is complementary to, but unique from, more traditional scientific research. As the name implies, qualitative research is designed to elicit a better understanding of patients’ lived experiences with a disease and disease-related factors and issues that are most important to them. This understanding is achieved not through formal instruments or quantitatively measured outcomes, but from the patients’ own words and vernacular. One of the most frequently used techniques in qualitative research studies is one-on-one interviews using a semi-structured, open-ended interview guide that provides for structure and consistency across interviews while allowing patients to express their specific thoughts and opinions. Thus, patients can freely discuss issues that are important to them and the study findings are primarily driven by patients’ narratives. Qualitative research provides a deeper context for understanding the “why” of patient experiences. For example, “Why was an experience positive or negative? Why do you feel this way? How has a therapy affected you and your HRQoL? What changes, if any, have you experienced with therapy that are meaningful to you?” The type of information gleaned from this type of research can be valuable to identify issues of high relevance to patients and which should be covered in HRQoL assessment tools.

While many clinical studies of HAE prophylaxis therapies have demonstrated significant improvements in one or more HRQoL domains [7–10], the specific reasons or context behind the improved scores have never been investigated using qualitative methods. Several of the authors (JA, DSL, WL, HHL) were involved in the COMPACT clinical trial program which evaluated the safety and efficacy of routine prophylaxis with subcutaneous C1INH (C1INH[SC]) in patients with HAE-C1INH. In the COMPACT trials, prophylaxis with C1INH(SC) was associated with measurable improvements from baseline in general health and anxiety, as well as reductions in work presenteeism, productivity loss, and activity impairment [8]. To further investigate these phenomena, a qualitative research study was designed to explore in-depth with real-world patients the reasons for improvements in HRQoL while using C1INH(SC) prophylaxis and to develop a better understanding of treatment attributes and benefits most important to patients. The study was also designed to understand whether there were concepts important to patients using HAE prophylaxis, in this case C1INH(SC), that were not included in the AE-QoL questionnaire, which is an angioedema-specific HRQoL instrument frequently used in HAE-C1INH research.

## Methods

This was a non-interventional, qualitative research study that involved patients identified through four HAE specialty practices in geographically separate regions of the United States (Alabama, California, Texas, Maryland). The study was exempted from ethics approval by the Chesapeake (currently, Advarra) IRB (Columbia, Maryland). Written informed consent was obtained from all patients prior to being interviewed.

### Patients

Each investigator invited patients to participate in an interview based on the following inclusion/exclusion criteria: patients had to be ≥ 18 years of age; diagnosed with HAE-C1INH type 1 or 2; be a native English speaker with the cognitive, linguistic, and social capacities necessary to participate in a 60-min phone interview; and current use of C1INH(SC) replacement therapy for ≥ 3 consecutive months. Individuals were excluded if they had any physical or mental conditions or substance abuse problems that might have interfered with their ability to participate in the study. Individuals currently enrolled in a clinical trial or any type of interventional study were also not eligible to participate.

### Interviews

Interviews were conducted by telephone between June 2018 and September 2018 by one of three interviewers from ICON plc, a global contract research organization. Each interviewer received project-specific training in qualitative interviewing and in maintaining patient confidentiality. The interviews were conducted following a non-scripted, semi-structured interview guide with open-ended questions to collect spontaneously reported information. If the patient being interviewed did not cover specific topics of interest, then the interviewer probed these topics as instructed in the interview guide. The interview guide (Additional file 1: Table S1) included topics that corresponded to the concepts covered by items in the AE-QoL. Covering the AE-QoL items in the guide enabled a comparison of concepts elicited during the interviews and those in the AE-QoL via a conceptual mapping correspondence analysis. The interview guide also included topics that AE-QoL items did not address to determine whether the AE-QoL sufficiently addressed concepts of interest to patients treated with C1INH(SC).

Each 1-h interview took place at a time agreed upon by each patient prior to the interview. At the completion of the interview, each patient received compensation of 100 USD in the form of a gift card for their participation. The interviews were audio recorded with the patients’ permission, transcribed verbatim by a third-party vendor, and anonymized to remove all personally identifiable information. Audio recordings were destroyed after analysis of the interviews was completed.

### Qualitative analysis

Qualitative analyses were conducted on all anonymized transcripts according to thematic analysis methods based on grounded theory principles [11]. Thematic analysis aims to identify themes and concepts that emerge from the data and, unlike other types of research, are not based on a priori hypotheses. Analysis was carried out using Atlas.ti software, version 8.0.

Analysis of transcripts yielded concepts and themes describing patients’ experience with HAE and C1INH(SC). A concept was defined as a lower-level category that described a specific symptom or impact. Concepts expressed by five or more patients (33% of the total sample of patients) were retained for analysis and grouped by themes. A theme was defined as a higher-level category of abstraction into which concepts were grouped by the ICON analyst. The end goal of the thematic analysis was to construct conceptual models illustrating themes and concepts important to patients with HAE who were using C1INH(SC). Each concept present in a conceptual model was given a definition and was described by illustrative quotes and by frequencies, i.e., the number of patients who spoke about the concept during the interview.

Two conceptual models were developed: (1) Impact of using C1INH(SC) prophylaxis on HRQoL related to symptom relief and (2) Use of C1INH(SC) compared to past HAE prophylaxis medications.

### Cross-mapping between interview concepts and AE-QoL questionnaire items

The interview guide was developed with the concepts contained in the AE-QoL in mind. As such, the interview guide topics covered the AE-QoL items. During coding of transcripts, when patients spoke about concepts that were consistent with the AE-QoL items, these were given code names that identified them as such.

In order to understand the conceptual model behind the AE-QoL, a targeted literature review was performed to find published articles about the questionnaire’s qualitative development. Once this was completed, the items of the AE-QoL were “mapped” one-by-one with the concepts in the conceptual model. This mapping exercise consisted of pairing the concept(s) with the item(s) that covered similar ideas. An Excel grid was used to document the mapping exercise.

## Results

Fifteen patients were recruited and interviewed from the four study sites. One patient was later determined to be ineligible when the patient reported diagnosis of acquired angioedema rather than HAE-C1INH type 1 or 2, leaving 14 patients in the analysis population. Table 1 provides a summary of the study population characteristics. The sample was diverse with respect to age (mean, 47.5 years; range, 28–72 years), sex (n = 9; 64.3% female), and time since HAE diagnosis (range, 6–61 years), but was limited with respect to ethnicity (n = 13, 92.9% white). A majority (n = 10; 71.4%) were employed either full-time or part-time. Comorbidities were self-reported in 9 (60%) subjects; comorbidities reported by more than one subject included allergic rhinitis or conjunctivitis/seasonal allergies, anxiety, depression, hypertension, and asthma. Most patients (n = 11; 78.6%) reported prior use of HAE prophylaxis (IV C1INH [C1INH(IV)] or androgens) before using C1INH(SC). Three patients initiated use of C1INH(SC) as part of a clinical trial, 10 started using it following FDA approval in June 2017, and one patient’s exact start date was unknown (but was > 3 months prior to the interview).

**Table 1:** Patient demographics and HAE treatment characteristics

	N = 14
Age, mean (range)	47.5 (28–72)
Gender, female, n (%)	9 (64.3)
Race, n (%)	
White	13 (92.9)
Black	1 (7.1)
Time since HAE diagnosis, years, mean (range)	31 (6–61)
Employment status, n (%)	
Full time	8 (57.1)
Part time	2 (14.3)
Retired	2 (14.3)
Full-time parent	1 (7.1)
Unemployed due to HAE	1 (7.1)
Comorbidities (self-reported), n (%)	
None	6 (40.0)
Allergic rhinitis/conjunctivitis or seasonal allergies	5 (33.3)
Hypertension	4 (26.7)
Anxiety	3 (20.0)
Depression	2 (13.3)
Asthma	2 (13.3)
GERD	1 (6.7)
Anemia	1 (6.7)
Hypothyroidism	1 (6.7)
Positive ANA nucleolar 1:640	1 (6.7)
Prior HAE prophylaxis	
Plasma-derived C1INH(IV)	9 (64.3)
Androgens	2^a^ (14.3)
No long-term prophylaxis	2 (14.3)
Unknown	1 (7.1)
Current on-demand treatment, n (%)	
Icatibant only	9 (64.3)
Icatibant and plasma-derived C1INH(IV)	2 (14.3)
Icatibant and recombinant C1INH(IV)	1 (7.1)
Plasma-derived C1INH(IV) only	1 (7.1)
Recombinant C1INH(IV) only	1 (7.1)

The above reflects information gathered at the time of interview

*C1INH(IV)* intravenous C1 esterase inhibitor, *GERD* gastroesophageal reflux disease, *HAE* hereditary angioedema

^a^1-stanozolol, 1-danazol

The analysis yielded an initial 24 themes and 205 concepts. When limited to concepts mentioned by at least one-third of patients (5 patients or more) or those mapped to an AE-QoL item (per protocol), a total of 16 themes and 38 concepts were included in two conceptual models, one pertaining to the impact of C1INH(SC) on HRQoL driven by symptom relief, and the other comparing C1INH(SC) to past HAE prophylaxis medications.

### Conceptual model #1: impact of C1INH(SC) on HRQoL

**Fig. 1:**
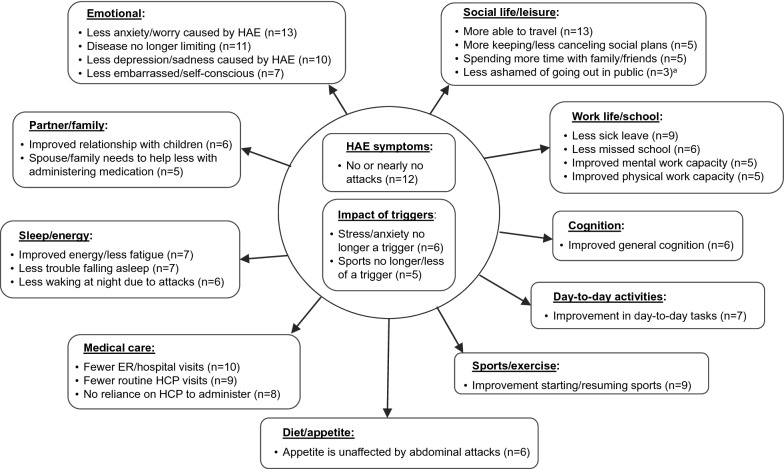
Conceptual model of HRQoL-related themes identified from interviews of 14 patients with HAE-C1INH using C1INH(SC) replacement therapy. The model included concepts which were identified by 5 or more patients during the interviews. Each box represents a theme; bulleted items are concepts. N values are number of patient interviews in which concept was mentioned. The conceptual model concluded that the themes of improved HAE symptoms and lessened impact of HAE attack triggers (center circle) impacted improvements in other themes. *AE-QoL* Angioedema Quality of Life, *ER* emergency room, *HAE* hereditary angioedema, *HCP* health care practitioner, *HRQoL* health-related quality of life, *C1INH* C1 inhibitor, *C1INH(SC)* subcutaneous C1INH. ^a^Concept included even though it was mentioned in less than n = 5 patient interviews because it relates to an item in the AE-QOL

Overall, this conceptual model illustrates the clinically relevant impact that C1INH(SC) replacement therapy had on reducing patients’ sensitivity to prior attack triggers (stress and anxiety; sports) and lessening the number of attacks to none or almost none. These changes in attack occurrence and trigger sensitivity led to improvement in a wide range of HRQoL domains depicted in the model. The conceptual model in Fig. 1 illustrates these improvements.

#### Changes in sensitivity to attack triggers

The conceptual model included the theme of reduced sensitivity to prior triggers of HAE attacks, such as stress or anxiety (n = 6) and sports (n = 5). The reduced frequency of HAE attacks triggered by sports meant that patients could practice sports more or resume sports they had previously stopped, notably high-impact sports, such as skiing. Certain high-impact physical activities could still trigger attacks in a few patients; the improvement they experienced while using C1INH(SC) was therefore limited to low-impact activities. One patient described not being able to horseback ride, bike, or lift weights, but she could do other types of sports: “As long as it’s like an elliptical or an aqua-aerobics class I can easily do that, yoga. But if it’s anything high impact causing trauma—even I took a weight training course and I ended up with swells all over, because you know, they push your muscles really hard” (patient 07-01).

Another prior HAE attack trigger for some patients was stress or anxiety. While C1INH(SC) treatment did not relieve the life stressors that were unrelated to HAE, it nonetheless made these stressors easier to bear. Patients no longer had to deal with both the impact of the HAE attack and the cause of the stressor. As patient 05–02 explained: “There’s still gonna be things that are gonna cause anxiety and cause stress and can lead to the possibility of an attack. It’s just that they, since I’ve been on [C1INH(SC)], they’re not happening. It’s just, it’s not getting to that point.”.

#### Changes in attack frequency

Twelve of the 14 patients described having no or nearly no attacks while using C1INH(SC); the other two patients spoke about experiencing a reduction in attack frequency on C1INH(SC). In this case, “nearly no attacks” was used to describe patients who had two or fewer attacks since starting treatment; patients who had only prodromal symptoms and then administered an acute treatment; or patients who had an attack because they missed a C1INH(SC) dose. The cutoff of “two or fewer attacks” since starting therapy emerged from the interviews as a general threshold for what patients considered “nearly no attacks.” A lowering of attack frequency could be the most important aspect of effective prophylaxis for some patients. In one patient’s words: “Since I started using it, I haven’t had any attacks. So that’s my main, my main thing. The others […] just didn’t work very well” (patient 07-02).

Beyond providing relief from the discomfort and pain of swelling, a reduction in the frequency of attacks or the absence of attacks had multiple impacts on patients’ HRQoL. The conceptual model of these impacts (Fig. 1) included ten themes related to HRQoL that were impacted: emotional life, work or school life, diet and appetite, day-to-day activities, partner/family life, medical care, sleep and energy, sports and exercise, cognition, and social life and leisure.

#### Emotional HRQoL

Almost all patients (n = 13 of 14) indicated during the interview that prophylaxis with C1INH(SC) improved their feelings of anxiety and worry caused by HAE. Patients were able to go about their lives as if “everything was under control” and without feeling as if “I’m waiting for the other shoe to drop” (patient 06-01). For two-thirds of patients (n = 10), this change in anxiety was accompanied by less reported depression due to HAE. Patient 01-02 described her life before C1INH(SC) as “dark” and “dreary.” Once her attacks stopped on C1INH(SC), she described her life as “energetic, fun, full, colorful.” Changes in feelings of depression and anxiety contributed to an overall feeling of being “normal” and “healthy” and not a “victim” of HAE. This change in perception was described by the concept of the “disease being no longer limiting” (n = 11). One patient stated: “I could, you know, do things like, you know, quote unquote a normal person can and not have those fears always in the back of my mind” (patient 06-03). Patients no longer felt embarrassed, self-conscious, or concerned about how other people might perceive any visible swelling and felt free to go out in public.

#### Work and school life

Many patients (n = 9) mentioned having to take less sick leave when on C1INH(SC). One patient (01-02) described being frequently hospitalized prior to starting C1INH(SC), and spending close to 200 days in the hospital during one year. Since starting C1INH(SC), she reported that she had gone four years without missing a single day of work due to HAE. None of the interviewed patients were currently in school, but some of them spoke about how HAE attacks kept them out of school in the past. A number of patients (n = 6) surmised that, if C1INH(SC) had been available at the time they had been in school, they would not have experienced HAE attacks and therefore could have avoided missing classes or attended classes without health complications.

In addition to reducing HAE-related missed work time, C1INH(SC) improved mental (n = 5) and physical (n = 5) work capacities. One patient described being able to “be there and feel well” while at the office and to “give 100%” to team projects with colleagues (patient 06-03). Patients were also able to plan and execute complex projects, like getting a new business off the ground (patient 05-01). Physical work capacity was important for both patients who worked in manual labor and patients who worked office jobs. In manual labor jobs, endurance and high-impact activities (like riding a tractor) would no longer trigger attacks. In office jobs, freedom from hand swelling meant patients could handle papers and pens and type on keyboards without hindrances.

#### Diet and appetite

A number of patients (n = 6) described improvement in appetite, mainly as a result of fewer abdominal attacks accompanied by pain and vomiting. Patients appreciated having a stable appetite that did not vary depending on whether they were having an abdominal attack, as described by patient 06-03: “I just don’t have that fluctuation, that up and down that I did previously. Then, again, going back to when I was sick all the time, I would go for periods where I couldn’t eat anything. I might just drink some liquids here and there to—then, after an attack, I’d be so hungry, I’d be like eating everything in sight. And it was kind of like just it went up and down when I was getting sick all the time.”

Some patients also mentioned regaining the ability to eat certain foods that in the past could have triggered HAE attacks. While this did not seem to influence improved appetite specifically, it did expand patients’ options when choosing what to eat.

#### Day-to-day activities

C1INH(SC) use enabled patients to have the energy and time to carry out day-to-day activities, such as grocery shopping and house cleaning (n = 7). Before C1INH(SC), when having attacks, patients needed to stop what they were doing for a while or not carry out those activities at all either because they were in so much pain or because they were embarrassed to have others see their swelling in public. Sometimes, a patient’s partner would have to take on the household tasks that the patient could not take care of alone. Furthermore, if patients were on IV prophylaxis medication before starting C1INH(SC), the time and planning it took to administer this medication impacted how a patient organized his or her day. In some instances, patients had to be dependent on others to administer medications. To be able to administer the medication themselves in the place and time of their choosing was liberating. One full-time parent who needed a nurse to administer her previous prophylaxis medication, explained it this way: “I’m a stay at home mom. I don’t work. I’m actually on disability because of the disorder because it was so bad a few years ago, it forced me to stop working. But I’m able, you know, to get more done around the house and I’m able to, you know, I go visit my dad a lot and go visit friends. I’m able to do that now. I cook and bake all the time, so I can get in the kitchen and don’t have to worry about, you know, time conflicts. Somebody showing up over here to give me an infusion. I go grocery shopping, you know, and do my errands every Friday. Before, I’d have to stop in the middle of that and come home, meet a nurse. I don’t have to worry about that now” (patient 07-02).

#### Family and partner relationships

Interview results indicated that C1INH(SC) use indirectly improved relationships between patients and their children (n = 6) and had benefits relating to less dependence on family members for help with drug administration (n = 5). For patients who required a partner’s help with IV prophylaxis medications in the past, C1INH(SC) gave the spouse back the free time they had lost and, moreover, relieved them of the sense of responsibility that they had felt previously. One patient said of her husband: “He’s not tied to me anymore that way. He’s not obligated” (patient 06-02). Patients with children were able to spend more time as a family and do activities together, such as sports, which were off limits in the past. One patient described how her HAE attacks interfered with vacations before using C1INH(SC): “Even my own family, my children, people would get very tired of me being sick. I was always sick. […] You know, we would make plans or, you know, we'd plan a vacation or—well, but there’s a summer we wanted to take my children to the beach. The first few days, I had to lay in a dark, cool [room] because I was so sick from an attack” (patient 01-01).

#### Time and expense related to medical care

Because C1INH(SC) reduced or eliminated attacks, it gave patients back time and money they previously spent on medical care. Having fewer attacks or no attacks translated into fewer emergency room (ER) visits and hospital stays for some patients (n = 10) and fewer routine visits to HAE physicians (n = 9). Less time in the hospital meant taking less sick leave, as well as patients and their families experiencing less stress and less financial strain. A decrease in the number of physician visits had similar impacts: patients who saw their physician every three to four months were now seeing them twice a year since their attacks were under control. This meant less travel time to and from doctors’ appointments and fewer co-pays. It also contributed to patients’ sense of being “in control of the disease” as well as being “normal” and “healthy.” Patient 06-01 stated: “It’s been really great. It’s almost like a blessing. Because Doctor [A]’s office is about an hour and a half to two hours away from where I live. So, the commute, all depending on the day and time, could be very frustrating because of traffic. […] It has really made a difference in, you know, just how I feel. You know, although I’ve had this disease pretty much all of my life, it just makes me feel like I really don’t have to kind limit myself for a lot of things I would not do because I was afraid of getting sick. Or it’s almost like I have more control over the disease that I’ve had all through my life.”

#### Sleep and energy

Changes in HAE attack burden had an effect on improving patients’ sleep and energy. Patients described HAE as interrupting sleep (trouble falling asleep or being awakened) because of the pain from the attacks and/or a fear of having potentially life-threatening throat swellings. As attacks stopped happening or happened less often, patients could fall asleep easier (n = 7) and sleep through the night more often (n = 6). Some patients started sleeping better once they were on C1INH(SC) because their anxiety levels decreased; previously, worries about having an attack, even if the patient had no symptoms at bedtime, would prevent patients from getting a good night’s sleep. As patients got more sleep and were less anxious, they experienced more energy and less fatigue (n = 7). One patient described being able to work the night shift at her job which would have been difficult for her before: “I actually switched back to the night shift in January […] I was worried about staying up all night, but I can. I don’t feel terrible […] I couldn’t do that before” (patient 01-04).

#### Sports and exercise

Being able to participate in sports without triggering an attack was an important change for many patients (n = 9). Prior to C1INH(SC), these patients had given up certain sports they were passionate about because of their disease. One patient was a devoted skier but had only gone skiing twice in 14 years before starting C1INH(SC). C1INH(SC) enabled him to resume skiing. Although he had a skiing accident and a serious knee injury, he was surprised and happy when he did not experience an expected HAE attack after this occurrence. He attributed the absence of an attack to his C1INH(SC) therapy. Later on, when he was on vacation, he was able to use crutches without an issue. Other described benefits included having to be less careful when exercising, having enough relief from pain and soreness to be able to exercise again, and being able to play sports with their children, as described by patient 01-04: “I used to want to play volleyball, you know. Your hands are going to swell immediately. But I actually played not too long ago and no swelling. I play with the kids and I taught them how to serve, and I can do that now.”

#### Cognition

A number of patients mentioned improvements in cognition since being on C1INH(SC) (n = 6). This was described as better memory and concentration and less “brain fog.” One patient who worked as a night technician in a sleep center said her thoughts were clearer and that she could remember patients’ faces better than when she was on androgens for her HAE (patient 01-04). Another patient described how, as her concentration improved, she was assigned more tasks at work: “I am more focused. I can get more things done. I’ve been added to some panels and some committees at work that I wasn’t in before because just my performance overall has improved” (patient 01-02). Another patient shared that he had experienced “brain fog” for so long that he did not remember what it was like to be without it: “I heard people talk about that brain fog and I didn’t know what that was. I didn’t realize what that was until I started C1INH(SC) and once not having that anymore, then I realized that’s what that was. I mean, that’s exactly how it is. It’s a lack of concentration. You can’t focus. It’s almost like you’ve taken some kind of cold medicine” (patient 01-01).

#### Social life and leisure

Treatment with C1INH(SC) improved patients’ social functioning. Patients described how they were able to enjoy their social lives more, including being able to spend more time with family or friends (n = 5) and being better able to plan ahead for social events without needing to cancel at the last moment because of an attack (n = 5). As discussed previously with regard to emotional factors, patients often felt more comfortable going out in public to socialize because they did not experience swelling (n = 3). However, for the large majority of patients (n = 13), the most important change C1INH(SC) made in their leisure time was that they could travel more freely. Before taking C1INH(SC) patients had to worry about finding a hospital or a physician if an attack occurred while away from home. With C1INH(SC) patients felt confident that the likelihood of having an attack was very low. Patients also felt comfortable traveling with C1INH(SC) and administering it on-the-go: in the car, in a restaurant, in a hotel room, or even while walking through an airport. Patient 01-02 explained how her approach to traveling changed dramatically: “I just pack a bag and go. I can go anywhere. I’ve been, I’ve been to Europe. I’ve been to the beach. I don’t have to worry about where’s the nearest hospital, will they know about my disease, will they know how to treat me. All of that used to be more important than what do I pack. Now the medicine is part of what I pack, but it’s not determining all of our plans.”

### Conceptual model #2: experience with C1INH(SC) vs past prophylaxis therapies

**Fig. 2:**
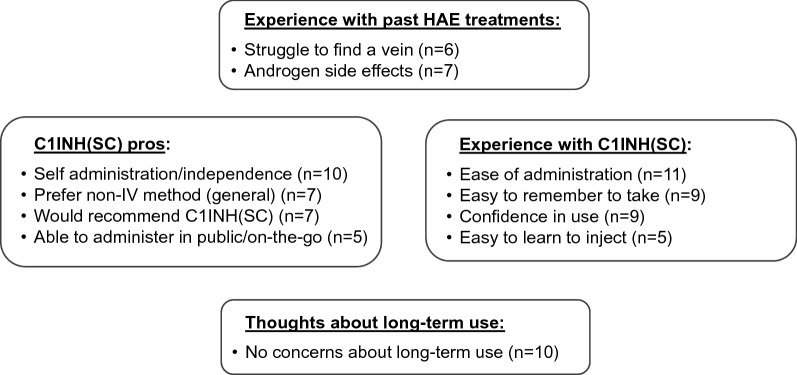
Patient opinions and experience with C1INH(SC) replacement therapy. Concepts and themes that emerged from 5 or more patient interviews out of N = 14 total interviews. *HAE* hereditary angioedema, *C1INH(SC)* subcutaneous C1 inhibitor

The second conceptual model, shown in Fig. 2, illustrates patients’ experience with the administration of C1INH(SC) and beliefs concerning using the medication long-term. This conceptual model should not be considered mutually exclusive from the first model on HRQoL benefits. Quite often, patients’ experience administering C1INH(SC) had an impact on the concepts in the conceptual model of HRQoL, in particular, concepts pertaining to the ability to travel and improvements in anxiety.

Prior to starting C1INH(SC), most patients had previously used other medication for HAE prophylaxis, including C1INH(IV) (n = 9) or oral androgens (n = 2). Each of these treatments had their own inconveniences or disadvantages. Patients who had used androgen therapy reported experiencing unpleasant side effects such as weight gain, mood swings, hair growth, deeper voices, depression, irregular menstrual cycles, and liver cancer. Patients who used IV prophylaxis reported struggling to find veins when administering the medication themselves and often requiring the help of a family member, a neighbor, or a nurse to inject the medication; one patient reported receiving infusions through a port. Even when patients managed to self-inject with IV injections, the repeated injections could be painful and lead to bruises and damaged veins. Patient 06-02 described what her experience was like when using C1INH(IV) for prophylaxis: “I looked like a junkie. I mean I have black and blue marks all the way up my arms and everything. I kept missing and then I hit the vein and then it would disappear.”

A majority of patients (n = 11) mentioned ease of administration as a benefit of C1INH(SC). Patients found the preparation and injection of C1INH(SC) to be easy to learn and easy to remember. One patient described learning to use C1INH(SC) in this way: “There was no further learning curve. I did it once and it was so easy to do that, oh, yeah, I’ve got this. I can totally do this. I can do it anywhere” (patient 05-01). Although a few patients expressed initial hesitation about switching to a SC medication, they quickly became confident using C1INH(SC) by themselves.

Half of the patients (n = 7) indicated during their interview that they would recommend C1INH(SC) to other HAE patients; interviewers did not systematically ask each patient if they would recommend C1INH(SC). There were three main advantages of C1INH(SC) administration from the patients’ perspective. First and foremost, patients appreciated being able to administer C1INH(SC) without assistance (n = 10), which facilitated feelings of control and independence. As patient 01–01 stated: “For the first time, I control my HAE and its treatment.” Second, there was an expressed general preference for non-IV mediation (n = 7), which reflected patients’ belief that IV medications were more difficult to administer and sometimes required the help of another person. Third, C1INH(SC) could be administered on-the-go, away from home, and even in public, if needed (n = 5). Patient 05-01 described using C1INH(SC) away from home: “If I’m at home, I’ll take it at home. Right? But I’ve also done it in the middle of the airport, and nobody even realizes what I’m doing. I’ve done it at a conference, at a table, where I put the syringe underneath the table. I don’t usually do it in the bathroom, although some people I’ve heard do that. I usually just find a table and an extra 20, 30 min, and I can do it wherever I am.”

A majority of patients expressed feeling comfortable about using C1INH(SC) long term, which was not the case with androgens or IV medications (n = 10). Patients said that since C1INH(SC) is a product derived from human plasma, they did not fear it would have side effects like androgens. In addition, patients said that because of C1INH(SC)’s SC administration, they no longer worried about not being able to find a vein.

### Most important benefits of C1INH(SC)

During interviews, each patient was asked to identify the most important benefit of C1INH(SC) from his/her point of view. Most of the responses reflected issues already included in the conceptual models (present in 5 or more interviews): ability to travel, keeping/not canceling social plans, ease of administration, absence of HAE attacks, less anxiety/worry about HAE, no longer feeling limited by HAE, preference for non-IV medication, and self-administration/independence. However, one additional concept that was cited as being the most important was the relief and/or gratitude that the patients’ children would have access to C1INH(SC). As HAE is a genetic disease, patients who were parents were often not only worried about their own health and HRQoL but also are concerned about those of their current children with HAE or their future children who might be born with the disease. Although only four patients mentioned this benefit, thus missing the cutoff of five patients for being factored into the conceptual models, it is mentioned here because of its importance and relevance to patients with children.

### Limitations of C1INH(SC)

While there were some limitations of C1INH(SC) therapy expressed during the interviews, none were mentioned by five or more patients, thus they were not captured in the conceptual models. The most commonly mentioned limitation was a preference for an oral treatment instead of a SC treatment (n = 4). Other limitations mentioned by two or more patients included: a desire for less frequent administration than the current twice per week dosing of C1INH(SC) (n = 2); initial apprehension to switch to SC from IV (n = 2; both of whom also described becoming quickly adapted to C1INH[SC] administration); and concern over the risk of scarred skin at injection site (n = 2). In addition, two patients mentioned concerns about drug supply; these patients had experienced a drug shortage while on C1INH(IV) in the past and were worried it could happen on C1INH(SC). None of the patients mentioned having experienced a shortage of C1INH(SC) while on therapy.

### Cross-mapping between interview concepts and AE-QoL questionnaire items

Table 2 displays the results of the cross-mapping exercise comparing the 38 concepts that emerged during five or more patient interviews against the AE-QoL questionnaire components.

**Table 2:** Cross-mapping of HRQoL concepts and themes identified from patient interviews with the AE-QoL questionnaire

Theme	Concept	AE-QoL coverage (AE-QoL item #)		
		Yes	Partially^a^	No
HRQoL-related concepts				
HAE symptoms	No or nearly no attacks			X
Change in impact of triggers	Stress/anxiety no longer a trigger			X
	Sports no longer/less of a trigger		2	
Emotional	Less anxiety/worry caused by HAE	13, 14		
	Disease no longer limiting	12		
	Less depression/sadness caused by HAE	10		
	Less embarrassed/self-conscious	3, 16		
Work life/school	Less sick leave	1		
	Less missed school		1	
	Improved mental work capacity		1	
	Improved physical work capacity	1		
Social life/leisure	More able to travel		3	
	More keeping/less canceling social plans		3, 4	
	Spending more time with family/friends		3, 4	
	Ashamed of going out in public	4, 15		
Partner/family	Improved relationship with children		2, 4	
	Spouse/family needs to help less with administering medications			X
Day-to-day activities	Improvement in day-to-day tasks		2	
Cognition	Improved general cognition		9	
Sleep/energy	Improved energy/less fatigue		8	
	Less trouble falling asleep	6		
	Less waking at night due to attacks	7		
Medical care	Less ER/hospital visits			X
	Less routine HCP visits			X
	No reliance on HCP to administer			X
Sports/exercise	Improvement starting/resuming sports		2, 3	
Diet/appetite	Appetite is unaffected by abdominal attacks		5, 11	
Treatment-specific concepts				
Experience with HAE treatments	Struggle to find a vein			X
	Androgen side effects			X
C1INH(SC) pros	Would recommend C1INH(SC)			X
	Self-administration/independence			X
	Prefer non-IV administration method			X
	Able to administer in public/on-the-go			X
Experience with C1INH(SC)	Ease of administration			X
	Easy to remember to take			X
	Confidence in use			X
	Easy to learn to inject			X
Long-term use	No concerns about long-term use	17		

Further detail on the cross-mapping between the AE-QoL questionnaire and patient interviews can be found in Additional file 2: Table S2

*AE-QoL* Angioedema Quality of Life, *C1INH(IV)* intravenous C1 esterase inhibitor, *ER* emergency room, *GERD* gastroesophageal reflux disease, *HAE* hereditary angioedema

^a^“Partially” indicates that AE-QoL items covering these topics were much more general/non-specific

All topics covered in the AE-QoL questionnaire emerged as concepts from the patient interviews (discussed by five or more patients). Nine concepts were issues/topics that are clearly addressed in the AE-QoL questionnaire: anxiety/worry; disease limitations; depression/sadness; embarrassment/self-conscious feelings; sick leave/work absence; physical work capacity; trouble falling asleep; waking up at night; and long-term medication safety. An additional ten concepts were considered to be partially addressed by AE-QoL questions: missed school time; mental work capacity; ability to travel; keeping and not canceling social plans; spending time with family and friends; relationships with children; general cognition; energy and fatigue levels; sports involvement; and appetite. There were 18 concepts identified in five or more of the patient interviews that were not addressed in the AE-QoL questionnaire, eight of which were HRQoL issues: absence of attacks; stress and anxiety no longer being triggers; sports no longer being a trigger; spouse or other family needing to help less with administering medication; improvement in ability to perform day-to-day tasks; fewer ER and hospital visits; fewer routine healthcare practitioner (HCP) visits; and no reliance on HCP to administer treatment.

A number of concepts that emerged were treatment-related concepts and, with the exception of “no concerns about long-term use” which is covered in general in AE-QoL question 17, other concepts within this group are not addressed in the AE-QoL (struggling to find a vein; side effects of androgens; feeling of confidence or independence because of self-administration; preference for non-IV mode of administration; ability to administer in public or on-the-go; ease of administration; being easy to remember to take or adherence to medication; confidence in use of medication; and ease of learning to use).

## Discussion

This study is the first known qualitative research project to identify concepts important to patients with HAE when using routine prophylaxis, in this case, C1INH(SC) replacement. The results build upon prior HRQoL research and provide in-depth insight on reasons for HRQoL improvements shown with HRQoL instruments in C1INH(SC) clinical trials [8]. The patients interviewed in this study reported greatly reduced frequency, or even a complete absence of attacks, which they attributed a wide variety of HRQoL improvements and new-found freedoms, including the following:

- Freedom from anxiety: almost all patients said C1INH(SC) use reduced their anxiety and worry about attacks and about giving themselves medicine intravenously. Improvement in depression was also common among patients.
- Freedom to use medicine independently, eliminating dependency on others: two-thirds of patients said that C1INH(SC) self-administration gave them back their independence. They no longer relied on nurses or family members’ help to deliver medication. Patients were free to plan their day as they saw fit and did not feel like a burden to others.
- Freedom to travel: almost all patients said that C1INH(SC) enabled them to travel more freely. C1INH(SC) could be transported and administered easily outside of the home. By preventing attacks, C1INH(SC) assuaged patients’ concerns about having an attack while away from appropriate medical care.
- Freedom from difficulties with administering medication: more than two-thirds of patients considered HAEGARDA easy to use compared to previous prophylaxis therapy, in particular IV medications.
- Freedom from their illness overall: for more than two-thirds of patients, C1INH(SC) made them feel as if their disease was no longer limiting their lives.
- Freedom from intensive medical care: for two-thirds of patients, C1INH(SC) use equaled less emergency room and hospital visits, which represented a financial and emotional relief for patients and their families. Furthermore, less time in the hospital meant more time for other activities. Nearly as many patients also spoke about visiting their HAE doctor less often, which provided a similar form of relief and meant less travel to and from the doctor’s office.
- Freedom to work unimpeded: for almost two-thirds of patients, C1INH(SC) use reduced the number of sick days patients had to take from work.
- Freedom from long-term concerns about medication: two-thirds of patients said they felt comfortable using C1INH(SC) long term, considering it safer than androgens or other nonhuman blood products.

The AE-QoL questionnaire is an HRQoL assessment tool used in HAE studies and which was developed using very similar qualitative methods as the current study. A cross-mapping exercise between the concepts that emerged from HAE patient interviews and items addressed in the AE-QoL confirmed that all of the items included in the AE-QoL were, in fact, identified as concepts in the patient interviews, thus confirming the relevance of the AE-QoL topics. However, a number of identified concepts represented topics that are not addressed by items in the AE-QoL, including sensitivity to potential attack-triggers (e.g., stress and anxiety, sports), attack frequency, not having to cancel social plans, ability to perform day-to-day tasks, and burden relating to frequency of medical visits (e.g., doctor, hospital, ER). Treatment-related concepts (e.g., ease of use and administration, preference, side effects, and satisfaction with treatment) identified in this study also appear to be important to measure from a patient perspective yet have not been widely incorporated into angioedema-specific HRQoL questionnaires. For example, the AE-QoL does not cover issues related to treatment, while the HAE-QoL does have a subscale for “treatment difficulties.” Further, neither the AE-QoL nor the HAE-QoL address issues of cognition, which were identified in the current study.

This study also identified a concept important to patients who have children: the anxiety over the potential effects and risks of HAE to the patients’ children, a phenomenon noted in prior research and interviews [12, 13]. This concept is not reflected in any generic HRQoL instruments, nor does the AE-QoL cover this aspect. It should be noted that the HAE-QoL, which is an HAE-specific instrument, does include a subscale for concerns about children.

A potential limitation to this study is that the sample size of 14 subjects could be considered small; however, populations of this size are fairly typical for qualitative research studies [14–20]. The cohort was almost entirely Caucasian and the findings may not be assumed to be generalizable for other racial groups. Another potential limitation is that patient invitation was at the discretion of the investigators rather than by random selection; however, random selection is not typically used for qualitative research.

## Conclusions

In summary, this is the first qualitative research study to obtain patients’ perspective and opinions about the use of prophylaxis therapy for HAE-C1INH. From these semi-structured interviews, a large number of common themes and concepts emerged in which patients shared the benefits they experienced relating to a greater sense of freedom, productivity, and improved personal relationships. These findings are not intended to imply that all patients who use C1INH(SC) will experience the benefits presented here. Rather, these qualitative data provide insights into the real-world experiences and factors which are important to patients with HAE-C1INH and highlight the multi-faceted HRQoL improvements that are likely possible with any convenient and effective prophylactic therapy. Future research of this type with other HAE therapies and more diverse subject cohorts would be of interest.

## Supplementary Information

**Additional file 1: Table S1.** Exploratory Interview Guide.

**Additional file 2: Table S2.** Cross-mapping details between AE-QoL questionnaire and patient interviews.

## Data Availability

Data sharing is not applicable to this article as no datasets were generated or analyzed during the current study. The information gathered and analyzed for this study was obtained from patient interviews which are not publicly available due to patient privacy reasons.

## Abbreviations

AE-QoL: Angioedema Quality of Life Questionnaire
HAE-C1INH: C1-inhibitor deficiency
ER: Emergency room
HRQoL: Health-related quality of life
HCP: Healthcare practitioner
HAE: Hereditary angioedema
HAE-QoL: Hereditary Angioedema Quality of Life Questionnaire
C1INH(IV): Intravenous C1INH
C1INH(SC): Subcutaneous C1INH
