# Supplementary material for: Letting the patients speak: an in-depth, qualitative research-based investigation of factors relevant to health-related quality of life in real-world patients with hereditary angioedema using subcutaneous C1 inhibitor replacement therapy
Source: Allergy Asthma Clin Immunol. 2021 Jun 27;17:60. doi: 10.1186/s13223-021-00550-5 (PMC8237414; doi:10.1186/s13223-021-00550-5)
Supplement: Supplementary file 1 — Additional file 1: Table S1. Exploratory Interview Guide. [file 13223_2021_550_MOESM1_ESM.docx]

**Additional file 1**

**Table S1.** Exploratory Interview Guide.

| **Concepts to Explore** | **Potential probes** |
| --- | --- |
| **Current Experience using C1INH(SC)** | |
| Satisfaction with C1INH(SC) therapy | - Please complete the following sentence: “The best thing about being treated with [C1INH(SC)] is __________________.” - Other patient needs that [C1INH(SC)] satisfies - Any fears about long-term effects of [C1INH(SC)]^a^ |
| How C1INH(SC) fits into the patient’s weekly routine | - - How patients remember to take the medication   - Where patients take the medication   - Time it takes patients to self-administer [C1INH(SC)]   - How confident patients feel taking the medication by themselves |
| Changes in medical care | - Frequency of visits to HCPs for treatment administration - Frequency of visit to HCPs for HAE routine health-care visits |
| Compare-contrast to experience with previous HAE prophylaxis medications, if any | - Main advantages of [C1INH(SC)] compared to previous preventive treatments, including route of administration (IV infusion vs. subcutaneous) - Any disadvantages of using [C1INH(SC)] |
| Patient perception of change in severity (location and frequency) of HAE attacks | - Changes in the location of attack (abdominal, urogenital, extremities, face, throat…) - Changes in the frequency of attacks - Changes in which attacks require acute treatment |
| **Impact of C1INH(SC) on health-related quality of life** | |
| Telling the story “My life now with [C1INH(SC)]” (creative exercise) | - - Ask patients to imagine that they were making a movie about their life with HAE before and after C1INH(SC)®   - Describe scenes/episodes before and after C1INH(SC)®, with details such as location, lighting, music, actors   - What is the main character (the patient) doing in these scenes |
| Compare and contrast before/after [C1INH(SC)]: impact of HAE on: |  |
| 1. Feelings about swelling episodes | - Changes in feeling anxious about having swelling episodes - Changes in feeling ashamed, embarrassed, or self-conscious about going out in public due to swelling |
| 2. Social and family life | - - Changes in time spent with and energy for friends and family, including ability to make and keep plans   - Changes in time spent on and energy for hobbies, including sports   - Changes in time spent on and energy for traveling   - Changes in fear about traveling and being away from home or a hospital or infusion center |
| 3. Physical well-being | - - Changes in ability to do basic physical activities in day-to-day life (walking short distances, using stairs, sitting/laying down, standing up…)   - Changes in ability to play or do more rigorous physical activities, such as running or walking long distances, playing sports |
| 4. Work and school life | - - Changes in ability to concentrate while at school/work^a^   - Changes in productivity at school or work, including working more/less hours   - Changes in not missing school or work because of HAE attacks   - Changes in ability to plan for career and educational opportunities in the long-term |
| 5. Day-to-day activities | - Changes in comfort level being outside of the house and driving (not worrying about an attack taking place) - Changes in time and energy for basic self-care (showering, getting dressed, eating…) - Changes in time and energy for household chores (cleaning, putting things away, laundry, dishes…) - Changes in time and energy for shopping and groceries |
| 6. Emotional well-being | - Changes in feelings of depression^a^ - Changes in your confidence in your ability to manage your disease - Changes in your ability to think and plan long-term - Changes in a lack of energy or fatigue - Changes in appetite - Changes in stress levels |
| 7. Eating and drinking | - - Changes in difficulties with swallowing   - Changes in your ability to eat and drink without difficulty   - Changes in pain when eating or drinking   - Changes in your freedom to choose foods and drinks |
| 8. Sleep | - Changes in difficulties falling asleep - Changes in waking up during the night and going back to sleep afterwards - Changes in feeling tired during the day because of lack of sleep |

All concepts were to be explored. Probes were optional (except for those footnoted as mandatory) and meant to be a way to stimulate discussion if the patient did not spontaneously bring up particular concept.

**^a^**Mandatory probes

HAE, hereditary angioedema; HCP, healthcare practitioners
