# Supplementary material for: Letting the patients speak: an in-depth, qualitative research-based investigation of factors relevant to health-related quality of life in real-world patients with hereditary angioedema using subcutaneous C1 inhibitor replacement therapy
Source: Allergy Asthma Clin Immunol. 2021 Jun 27;17:60. doi: 10.1186/s13223-021-00550-5 (PMC8237414; doi:10.1186/s13223-021-00550-5)
Supplement: Supplementary file 2 — Additional file 2: Table S2. Cross-mapping details between AE-QoL questionnaire and patient interviews. [file 13223_2021_550_MOESM2_ESM.docx]

**Additional file 2**

**Table S2**. Cross-mapping details between AE-QoL questionnaire and patient interviews.

| **AE-QoL item** | **Corresponding qualitative concept(s) from interviews** | **Comments** |
| --- | --- | --- |
| **Instructions for AE-QoL items #1-5: Indicate how often in the last four week you have been restricted in the areas of your daily listed below because of swelling episodes (angioedema). (Regardless of whether or not you have actually experienced swelling episodes during the time period)** | | |
| 1. Work | "Physical work capacity"; "Mental work capacity," "Missing school"; "Missing work" | It is difficult to determine what specific concepts patients will think about when answering these items because they are very broad. Depending on how a patient interprets an item, multiple concepts may be considered when an item is answered. In order to be exhaustive, therefore, multiple concepts are suggested for each item |
| 2. Physical activity | "Start/resume sports"; "Relationship with children"; “Day-to-day tasks"; “Sports no longer a trigger" |  |
| 3. Leisure time | "Able to travel"; “Ashamed of going out in public"; “Spending time with friends/family"; “Keeping/not cancelling social plans"; “Embarrassed/self-conscious"; “Start/resume sports”; “Sports no longer a trigger” |  |
| 4. Social relations | “Ashamed of going out in public"; “Spending time with friends/family"; “Keeping social plans"; “Embarrassed/self-conscious"; “Relationship with children" |  |
| 5. Eating and drinking | "Appetite is unaffected by abdominal attacks" | It is uncertain if patients will think about the affect of abdominal attacks on appetite when answering this item because it does not specifically ask about this impact. |
| **Instructions for AE-QoL items #6-17: In the following questions we would like to get more details about the difficulties and problems that may be associated with your recurrent swelling episodes (angioedema) (during the last 4 weeks)** | | |
| 6. Do you have difficulty falling asleep? | “AE-QoL: Trouble to fall asleep" | These items are directly related to concepts from interviews |
| 7. Do you wake up during the night? | “AE-QoL: Wake up at night because of an attack" |  |
| 8. Are you tired during the day because you are not sleeping at night? | "Energy/fatigue" | Patients are tired because they do not sleep well at night, but also because they are stressed/anxious. The item does not distinguish between these two sources of fatigue. |
| 9. Do you have trouble concentrating? | "Cognition: general" | Patients speak about improvements in concentration/focus, but also about other cognitive improvements in brain fog or memory. |
| 10. Do you feel depressed? | "Depression/sadness caused by HAE" | Patients do not always use the word "depression"; some say "sad.” |
| 11. Do you have to limit your choices of food or beverages? | "Appetite is unaffected by abdominal attacks" | This item overlaps with item #4. It is unclear if patients will think of abdominal attacks when answering this item given how it is phrased. |
| 12. Do your swelling episodes place a burden on you? | "Disease is no longer limiting" | The word “burden” is may be interpreted in many ways by patients. |
| 13. Are you afraid that a swelling episode could occur suddenly? | "Anxiety/worry" | Patients do not distinguish between the two sources of anxiety included in AE-QoL items #13 and #14. |
| 14. Are you afraid that the frequency of the swelling episodes might increase? | "Anxiety/worry" |  |
| 15. Are you ashamed to go out in public because of the swelling episodes? | “AE-QoL: Ashamed of going out in public" | These items are directly related to concepts from interviews. |
| 16. Do the swelling episodes make you embarrassed or self-conscious? | “AE-QoL: Embarrassed/self-conscious" |  |
| 17. Are you afraid that the treatment of the swelling episodes could have negative long-term effects for you? | “AE-QoL: No long-term concerns" |  |
